# Cervical stenosis following electrosurgical conization

**DOI:** 10.1590/S1516-31802008000400002

**Published:** 2008-07-03

**Authors:** Aparecida Cristina Sampaio Monteiro, Fábio Bastos Russomano, Maria José de Camargo, Kátia Silveira da Silva, Fernanda Rangel Veiga, Rebecca Guimarães Oliveira

**Keywords:** Electrosurgery, Cervical intraepithelial neoplasia, Postoperative complications, Conization, Constriction, pathologic, Eletrocirurgia, Neoplasia intra-epitelial cervical, Complicações pós-operatórias, Conização, Constrição patológica

## Abstract

**CONTEXT AND OBJECTIVE:**

Cervical stenosis is a postoperative complication of procedures for treating preinvasive lesions of the cervix and takes on particular importance due to the clinical repercussions associated with it. Furthermore, it causes limitations in relation to cytological and colposcopic follow-up. The aim here was to assess the incidence of cervical stenosis among a cohort of patients who underwent electrosurgical conization and to identify possible prognostic factors associated with its occurrence.

**DESIGN AND SETTING:**

Retrospective study at Gynecology and Obstetrics Department, Instituto Fernandes Figueira, Rio de Janeiro.

**METHODS:**

This was an observational study among a cohort of patients who underwent electrosurgical conization of the uterine cervix. The possible predictive variables were analyzed as bivariate means between the groups with and without stenosis. We also calculated the incidence density rate ratio for cervical stenosis in relation to each possible predictive variable and the respective confidence intervals (95%). Levels of 5% were considered significant.

**RESULTS:**

274 patients who underwent electrosurgical conization of the uterine cervix with a minimum follow-up period of six months were included. The crude incidence of cervical stenosis was 7.66% and the incidence density was 3.3/1,000 patients-month.

**CONCLUSIONS:**

We did not find associations between the variables for stenosis. However, we observed borderline significance levels relating to hemorrhagic complications before and after the operation (p = 0.089).

## RESUMO

**CONTEXTO E OBJETIVO:**

A estenose cervical é uma complicação pós-operatória dos procedimentos para tratamento das lesões pré-invasivas do colo uterino e assume importância particular tanto pelas repercussões clínicas associadas como pela limitação causada nos acompanhamentos citológico e colposcópico. O objetivo foi verificar a incidência da estenose cervical em uma coorte de pacientes submetidas a conização eletrocirúrgica do colo uterino e apontar possíveis fatores prognósticos associados.

**TIPO DE ESTUDO E LOCAL:**

Estudo retrospectivo, observacional realizado no Instituto Fernandes Figueira, Departamento de Ginecologia, Rio de Janeiro, Brasil.

**MÉTODOS:**

Estudo observacional em uma coorte de pacientes submetidas à conização eletrocirúrgica do colo uterino. As variáveis possivelmente preditoras foram analisadas de forma bivariada entre os grupos com e sem estenose. Calculou-se também a razão de taxas de incidência-densidade da estenose cervical para cada variável possivelmente preditora e os respectivos intervalos de confiança (95%). O nível de significância considerado foi de 5%.

**RESULTADOS:**

Foram incluídas 274 pacientes submetidas a conização eletrocirúrgica do colo uterino com um tempo mínimo de seis meses de acompanhamento após o procedimento. A incidência bruta de estenose cervical foi de 7,66% e a incidência-densidade foi de 3,3/1000 pacientes-mês. Não houve diferença estatisticamente significativa entre as características clínico-demográficas dos grupos.

**CONCLUSÃO:**

Não se encontrou associação entre as variáveis e a estenose, porém observou-se um nível de significância limítrofe em relação às complicações hemorrágicas do peri e pós-operatório (p = 0,089).

## INTRODUCTION

Cervical stenosis consists of partial or complete obstruction of the cervical canal and it is considered to be one of the most important late complications of laser cone biopsy.^1^

The increasing number of young women of reproductive age presenting preinvasive lesions of the cervix has made it necessary to treat such lesions more conservatively.^2^ It cannot be asserted that the incidence of preinvasive lesions is increasing, but on the other hand, the diagnostic practices directed towards these diseases today feature greater sensitivity. Thus, it has become possible for greater numbers of cases to be diagnosed.^3^ Electrosurgical excision techniques have now become more widespread and are considered to be a more conservative form of treatment. However, the procedure is not exempt from morbidity. The most common complications are pre and postoperative hemorrhages, infections, cervical stenosis, fertility issues and pregnancy-related complications.^4^

Cervical stenosis takes on particular importance because of the clinical repercussions that are associated with its occurrence, such as dysmenorrhea, amenorrhea, infertility and lesions during labor. Furthermore, it causes limitations in relation to the cytological and colposcopic follow-up after treatment for preinvasive cervical diseases, thereby making it difficult for residual or recurrent diseases to be diagnosed.^5,6^

There is no consensus in the literature regarding the definition of cervical stenosis. Because of the different definitions used, the incidence observed by each author has also varied (from 0 to 25.9%).^7-10^ Better knowledge regarding this complication of electrosurgical conization of the cervix would enable the planning of prevention strategies.

## OBJECTIVE

The purpose of this study was to assess the incidence of cervical stenosis among a cohort of patients who underwent electrosurgical conization of the cervix in the Cervical Pathology and Colposcopy Unit of the Department of Gynecology, Instituto Fernandes Figueira (IFF), Fundação Oswaldo Cruz (Fiocruz), in the city of Rio de Janeiro. An additional aim was to point out possible prognostic factors associated with its occurrence.

## MATERIALS and METHODS

This observational study dealt with a cohort of patients who underwent electrosurgical conization of the cervix between January 1998 and May 2006. The outcome of interest was the occurrence of cervical stenosis following electrosurgical conization, along with the further variables that were possibly associated with its occurrence. The sample size calculated was 270 patients, taking a precision of 95%. We included all patients who underwent electrosurgical conization of the cervix with postoperative follow-up of at least six months. This period included the first appointment after the treatment at which a diagnosis of stenosis could be established.

For the electrosurgical conization, we used a straight wire or diathermy loop, according to the surgical technique used, as described by Prendiville.^11^ When we used a loop, the technique used was large-loop excision of the transformation zone-cone (LLETZ-cone), while the technique was straight-wire excision of the transformation zone (SWETZ) when we performed conization using a straight wire of at least 5 mm in length. The follow-up after treatment included cytological and colposcopic examinations every six months for a maximum period of 12 to 24 months, in accordance with the routine established by our Unit.

The data were stored in a database (Microsoft Access 1997 format) in the Cervical Pathology and Colposcopy Unit of our institution and the information was acquired by means of reviewing data forms.

The diagnosis of cervical stenosis was established when a clinically relevant partial or complete obstruction of the cervical canal was observed that made it impossible to reach the endocervical cells using a cytobrush, guided by colposcopy. It was also established when associated with a clinical complaint of significant secondary dysmenorrhea following conization, or the presence of an echographic image suggestive of hematometra, thereby making it necessary to dilate the cervix.

The predictive variables evaluated were: age at the time of conization; absence of vaginal delivery in the obstetric history; menopause; another electrosurgical procedure before or after the conization (history of LLETZ before or after conization, electrocauterization of the cervix before conization, or reconization of the cervix); type of surgical technique used for the electrosurgical conization of the cervix (LLETZ-cone or SWETZ); volume of the surgical specimen; more than one surgical specimen; and presence of hemorrhagic complications during the immediate pre or postoperative period. We calculated the cumulative incidence, the incidence density of cervical stenosis and the estimated likelihood of cervical stenosis over time using Kaplan-Meyer method, taking this to be an open cohort in which patients had different lengths of follow-up. The possible predictive variables were analyzed using bivariate analysis, in relation to the outcome. This was done in conjunction with the chi-squared test and Fisher exact test, whenever it was necessary to test differences relating to proportions, and with Student's t test for differences regarding mean continuous variables. We also calculated the rate ratio for the incidence density of cervical stenosis for each possible predictive variable, with 95% confidence intervals, using the statistical analysis software Epi-Info version 6.04. The Statistical Package for the Social Sciences (SPSS) version 8.0 was used for the remaining analyses. The significance level was taken to be 5%.

This study was approved by the Research Ethics Committee of IFF.

## RESULTS

Up to May 31, 2006, 274 patients were included in this cohort and underwent electrosurgical conization of the cervix with a minimum period of six months follow-up after the procedure. The cumulative incidence of cervical stenosis following electrosurgical conization in this cohort was 7.66%, and the incidence density was 3.3/1,000 patients-month. The characteristics of the groups with and without stenosis are described in Table 1. We did not observe any statistically significant differences between the groups.

**Table 1:** Clinical and demographic characteristics of the patients included in the cohort (Instituto Fernandes Figueira, 2006)

Variables	Whole cohort (n = 274)	Cervical stenosis n = 21 (7.66%)	Normal cervix n = 253 (92.34%)	p-value
**Patients**				
Mean age in years at the time of conization (SD)	44.60 (10.05)	47.13 (11.30)	44.35 (9.93)	0.223*
Postmenopausal: n (%)	60	7 (33.33)	53 (21.37)	0.272†
HIV-positive: n (%)‡	37	3 (16.67)	34 (15.89)	1.00†
Absence of vaginal delivery: n (%)	45	2 (9.52)	43 (17.34)	0.544†
Previous LLETZ: n (%)	11	2 (9.52)	9 (3.56)	0.202†
Previous electrocauterization: n (%)	2	0	2 (0.79)	–
LLETZ following conization: n (%)	2	0	2 (0.79)	–
Reconization: n (%)	3	0	3 (1.18)	–
**Procedures**				
LLETZ-cone: n (%)	171	10 (47.62)	161 (63.64)	0.145§
SWETZ: n (%)	103	11 (52.38)	92 (36.36)	
Pre or postoperative hemorrhagic complications: n (%)	9	2 (9.52)	7 (2.77)	0.145†
**Specimens**				
Mean volume of specimen in cm^3^ (SD)	10.81 (12.66)	14.85 (21.20)	10.37 (11.48)	0.133*
**Fragmentation of the specimen**				
One fragment: n (%)	222 (81.9)	18 (85.72)	204 (81.60)	0.775†
More than one fragment: n (%)	49 (18.1)	3 (14.28)	46 (18.40)	

* *Student's t test;*

† *Fisher's exact test;*

‡ *43 patients (15.7%) refused to take the HIV-test;*

§ *chi-squared test.*

*SD = standard deviation; LLETZ = large-loop excision of the transformation zone; SWETZ = straight-wire excision of the transformation zone.*

**Figure 1:**
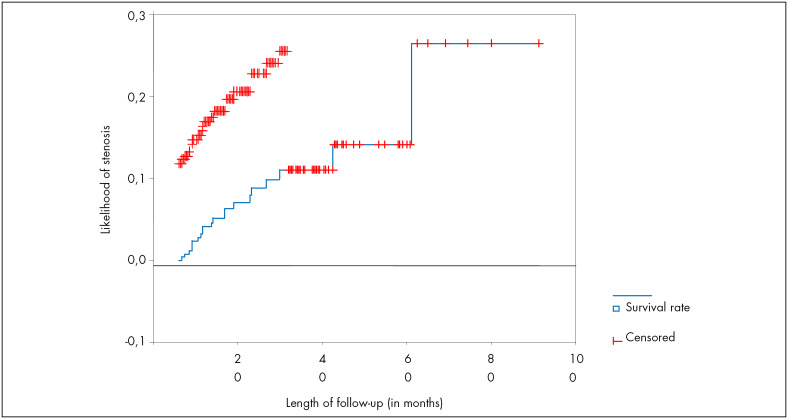
Likelihood of cervical stenosis over time (Instituto Fernandes Figueira, 2006).

To evaluate the likelihood of cervical stenosis during the follow-up, we used the Kaplan-Meyer method (Figure 1).

For each possible predictive variable, we estimated the rate ratio for the incidence density of cervical stenosis, taking into consideration the summed duration of follow-up for each patient in the denominator (Table 2).

**Table 2:** Risk of cervical stenosis according to variables (Instituto Fernandes Figueira, 2006)

Variable	Cases of stenosis (n)	Length of follow-up	p-value*	Rate ratio	Confidence interval (95%)
Age†			0.26		0.67-4.11
Older than 43 years	14	3493		1.66	
Up to 43 years	7	2898		1	
Postmenopausal			0.30		0.65-3.96
Yes	7	1522		1.66	
No	14	4869		1	
Vaginal delivery			0.24		0.10-1.85
No	2	1252		0.43	
Yes	19	5139		1	
Previous LLETZ			0.40		0.43-7.92
Yes	2	345		1.84	
No	19	6046		1	
Previous electrocauterization – n (%)	Could not be evaluated because of the presence of zero values				
Type of procedure			0.37		0.62-3.46
SWETZ	11	2738		1.47	
LLETZ-cone	10	3653		1	
Yes	2	208		3.3	
No	18	6183		1	
Volume of the specimen (cm^3^)†			0.22		0.25-1.40
Greater than 7	9	3573		0.59	
Up to 7	12	2818		1	
Fragmentation of the specimen			0.8		0.25-2.91
Yes	3	1020		0.86	
No	18	5251		1	

* *chi-squared test;*

† *The cutoff points for the continuous variables of age and volume were obtained through calculating the mean for the population in the cohort.*

*LLETZ = large-loop excision of the transformation zone; SWETZ = straight-wire excision of the transformation zone.*

Table 3 presents the relevant clinical characteristics following the conization.

**Table 3:** Relevant clinical characteristics following conization (Instituto Fernandes Figueira, 2006)

Variable	Cervical stenosis (%)	Normal cervix (%)
Satisfactory cytology*		
Yes – n (%)	2 (9.52)	221 (91.33)
No – n (%)	19 (90.48)	21 (8.67)
Satisfactory colposcopy†		
Yes – n (%)	–	66 (100)
No – n (%)	21 (10.88)	172 (89.12)
Secondary dysmenorrhea		–
Yes – n (%)	4 (19.05)	
No – n (%)	17 (80.95)	
Hematometra		–
Yes – n (%)	4 (19.05)	
No – n (%)	17 (80.95)	
Dilation of the cervix –Yes (% in the group with stenosis)	8 (38.1)	–

* *Cytology was considered satisfactory when endocervical cells were present. The data relating to the adequacy of cytology were collected from 263 data forms only;*

† *Colposcopy was considered satisfactory when the transformation zone was completely visible. In 15 patients, it was not possible to find such data or they did not undergo colposcopy during follow-up consultations.*

## DISCUSSION

The incidence density obtained was 3.3 cases per 1,000 woman-months. We chose this measurement because, in this open cohort, each patient had a different length of follow-up. The articles published so far make reference to cumulative incidence, and in this cohort it was found to be 7.66%, thus not surpassing the percentage described in literature when electrosurgical conization was performed^8,10,12,13^ (Table 4), which ranged from three to 25.9%. This makes it difficult to compare the frequencies of cervical stenosis, because the definition of stenosis varies greatly among the studies.^10^

**Table 4:** Articles that refer to cervical stenosis following conization (Medline 1986-2005)

Authors, year	Total population and study design	Definition of cervical stenosis	Procedure reported in the study	Incidence of stenosis found (%)	Time at which the stenosiswas diagnosed	Prognostic factorfor stenosis	Studylimitation
Baldauf et al., 1996^10^	532 patients who underwent conization (laser or electrosurgery) – retrospective observational study	Narrowing that prevented insertion of a 2.5 mm Hegar dilator into the cervical canal	Laser cone (n = 255)LLETZ-cone (n = 277)	10.2 4.3	94.7% of cases within the first six months.	Endocervical lesion, RR: 4.10 (95% CI: 1.75 – 9.61) Height of cone ≥ 20 mm, RR: 4.33 (95% CI: 1.57-11.92)	Doubt regarding whether the length of follow-up for the LLETZ-cone group was enough to diagnose all the cases of stenosis.
Ferriset al.,1995^12^	198 patients who underwent electrosurgical procedures. Multicenter prospective cohort	Inability to pass a small cotton-tipped swab into the endocervical canal	LLETZ LLETZ-cone	3.8 25.9	Not described.	LLETZ-cone, RR 5.65 (95% CI: 1.35-23.69) Height of cone ≥ 10 mm, p = 0.002	31% of patients were lost from follow-up.
Suh-Burgmannet al.,2000^1^	164 patients who underwent to electrosurgical procedures Retrospective observationalstudy	Requirement for dilation with an endocervical curette of 3.0 mm diameter to collect endocervical samples	Procedures with diathermy loop	6	Not described. Follow-up lasting one year.	Volume of the excised tissue greater than 6.6 mm^3^, RR 1.32 (95% CI: 1.1-1.67)Previous history of electrosurgical procedure	Was follow-up period long enough?
Houlardet al.,2002^9^	238 patients who underwent to laser conization. Prospective study	Cervical narrowing preventing insertion of a 4.0 mm diameter cotton swab into the cervical canal	Laser cone	16.8	Not specified. Total length of follow-up of 26 months.	Age > 40 years RR 4.95 (95% CI: 1.8-8.6)	
Brunet al.,2002^13^	241 patients who underwent conization. Retrospective observational study	Inability to introduce a 2.5 mm Hegar dilator into the cervical canal	Cold knife (n = 100); Laser cone (n = 39); LLETZ-cone (n = 102)	8 27 3	First 12 months after operation.	Laser cone p < 0.001	Was follow-up period enough? Other prognostic factors were not tested.
Mathevetet al.,2003^8^	86 patients who underwent conization (cold knife, laser, and LLETZ-cone). Randomized clinical trial	Inability to introduce a 3.0 mm Hegar dilator into the cervical canal	Cold knife (n = 37); Laser (n = 37); LLETZ-cone (n = 36)	14.3 0 3.4	First six months after surgery. Total length of follow-up of 36 months.	Cold knife, p = 0.03 and 0.06 when compared with laser and LLETZ-cone, respectively. Volume of the cone ≥ 2.1 cm^3^, p = 0.001	Suggestion that a study with a bigger sample size should be conducted to prove results. The method for blinding of envelopes was not explained.
Pennaet al.,2005^7^	1218 patients who underwent laser conization, 7.8% after menopause Retrospective observational study	Cervical narrowing that prevented the insertion of a 2.5-3 mm Hegar dilator to collect endocervical cytology	Laser cone	7.1	First six months after surgery.	Hormone replacement therapy was a protective factor OR 4.82 (95% CI 1.45-16.08)	Significant difference between the groups when nulliparity and endocervical lesion were compared.

*LLETZ = large loop excision of the transformation zone;*

*RR = relative risk; CI = confidence interval; OR = odds ratio.*

Ferris et al.^12^ suggested that the more liberal the parameter used to define stenosis is, the greater the incidence will be. They believed that this had been shown in their study.

Kaplan-Meyer curve analysis (Figure 1) suggested that the stenosis was evenly distributed, i.e., there was no relationship between higher frequency of the outcome and any given time. The likelihood of the occurrence of cervical stenosis after the 61^st^ month of follow-up was 26.45% (confidence interval, 95% CI: 3.21-49.6). However, this measurement may be imprecise because the number of patients undergoing follow-up at that time was rather small. In line with data from the authors cited in Table 4, the majority of the stenosis cases were diagnosed up to one year after surgery. Nevertheless, we cannot assert that the total length of follow-up described in the studies by Suh-Burgmann et al.^1^ and Brun et al.^13^ was enough to diagnose the outcome for all the cases.

Patients older than 43 years of age presented a 1.6 times greater chance of developing cervical stenosis, compared with the group of younger age. The same value was found when we analyzed the risk of stenosis in relation to the menopause. However, these variables did not reach a statistically significant difference (p = 0.26 and 0.30, respectively). Houlard et al.^9^ found that age greater than 40 years was a risk factor for cervical stenosis after laser conization (relative risk, RR = 4.95; 95% CI: 1.8-8.6). When the age cutoff point was 40 years, as in the study by Houlard et al.,^9^ patients over 40 years old presented a 1.48 times greater chance of developing cervical stenosis, compared with the younger group. Nonetheless, no statistically significant difference was observed (p = 0.68). Penna et al.^7^ evaluated age, parity, menopause, time of menopause, hormone replacement therapy (HRT) and previous surgical procedures in the cervix as risk factors for cervical stenosis following laser conization. All these variables were studied in relation to a comparison between postmenopausal women (mean age of 53 years) and women of reproductive and fertile age (mean age of 31 years). They concluded that the overall incidence was significantly higher in the postmenopausal group (p < 0.005). Moreover, they declared that HRT was the sole factor relating to a lower risk of postoperative cervical stenosis (odds ratio, OR 4.82; 95% CI: 1.45-16.08). Nonetheless, only 7.8% of the women were postmenopausal and the population of this group varied significantly with regard to parity and endocervical disease.

We evaluated whether vaginal delivery was absent from the subjects’ obstetric history and found a rate ratio of 0.43. This meant that if the patient had not had a vaginal delivery, the risk of cervical stenosis seemed to be diminished, despite the absence of statistically significant differences between the groups. We expected that ripening of the cervix during vaginal delivery might reduce the risk of stenosis, but we were unable to observe whether a lack of vaginal delivery in the patient's obstetric history was related to cervical stenosis. The value found created the assumption that there might have been some confounding factor, and therefore we wondered whether the patients who had had vaginal deliveries had also presented some other factor that might relate to the occurrence of cervical stenosis. To evaluate this possibility, we performed a stratified analysis (data not shown) in relation to vaginal delivery, age and height of cone, but we were unable to prove the existence of confounding. Baldauf et al.,^10^ Suh-Burgmann et al.^1^ and Penna et al.^7^ evaluated the risk of cervical stenosis following conization and found that nulliparity was not a prognostic factor for stenosis, with relative risks of 1.19 (95% CI: 0.39-3.65), 0.52 (95% CI: 0.14-1.93) and 1.64 (95% CI: 0.44-6.19), respectively for these three studies. For the nulliparous women, the result found by Suh-Burgmann et al.^1^ was similar to the findings of the present study: RR 0.52 (95% CI: 0.14-1.93), but without statistical significance.

We considered that a history of another surgical procedure in the cervix could be related to a higher risk of cervical stenosis, and therefore LLETZ, electrosurgical cauterization of the cervix prior to conization, LLETZ after conization and reconization were investigated. The latter three variables could not be tested because they featured a zero value in one of the categories. The risk of stenosis among patients who underwent LLETZ prior to conization was 1.84 (95% CI: 0.43-7.92), but the difference was not statistically significant. A history of previous LLETZ was a significant risk factor for cervical stenosis in the study by Suh-Burgmann et al. (op. cit.)^1^ (OR 17.4; 95% CI: 1.7-112), but they considered that they had a low number of cases with stenosis, and the relationship between previous surgical procedures and cervical stenosis was unclear. They suggested that repeated trauma or the sum of the previous excision with the latest conization would result in a greater volume of tissue removed, which might be responsible for the occurrence of stenosis.

Regarding the surgical techniques used for conization in the Cervical Pathology and Colposcopy Unit of IFF, the risk of cervical stenosis was 1.47 times greater (95% CI: 0.62-3.46) when SWETZ was applied. In spite of the absence of a statistically significant relationship, we expected that this technique might be related to a higher volume of tissue removed and consequently would be related to a higher likelihood of stenosis. All the same, we could not prove such an association (p = 0.37). For the variable of hemorrhagic complications during the immediate pre or postoperative period, the risk of cervical stenosis was 3.3 times higher (95% CI: 0.77-14.23; p = 0.089), compared with the procedure without complications. Despite the fact that we still could not prove a statistically significant difference, the borderline significance level allowed us to suggest that there may be a relationship between this variable and cervical stenosis. We did not find any studies in literature referring to any relationship between cervical stenosis and the two techniques cited in this study comparatively, or even any studies correlating excessive bleeding to cervical stenosis.

The volume of the excised surgical specimen could be calculated from its dimensions, in accordance with calculations described in pathological anatomy reports. In line with other authors,^1,8^ we believe that greater volume could relate to the occurrence of stenosis. However, we obtained a paradoxical result and we suppose that there may have been some other factor causing a confounding effect, but on the other hand, the stratified analysis correlating volume, age and surgical technique did not prove this possibility. We also hoped that the variable of more than one surgical specimen might represent a greater volume of excised tissue, thus implying an association with the outcome. In fact, this variable did express the volume and thus provided this result. In the study by Suh-Burgmann et al. (op. cit.),^1^ the volume of tissue removed during conization was considered to be a prognostic factor for cervical stenosis (OR 1.32; 95% CI: 1.1-1.67). However, they considered that their small sample of cases was a limiting factor (n = 10, out of 164 patients). Luesley et al.^5^ stated that cervical stenosis was more common in patients who had higher cones (more than 2.5 cm height) and proposed that the height of the cervical canal to be removed should be measured for the size of the cone, in order to be as small as possible, thus reducing the complications.

Brun et al.^13^ compared conization by three techniques: cold knife, laser cone and LLETZ-cone, and they recommended using a diathermy loop, because they considered that this technique removes a smaller volume of tissue and thus is related to a lower frequency of stenosis. However, they did not investigate any other prognostic factors for stenosis. According to Houlard et al.,^9^ there is a tendency for stenosis to occur when the cone height is greater than 20 mm (1.9; 95% CI: 0.9-4.1). Baldauf et al.^10^ found a relative risk of 4.33 (95% CI: 1.57-11.92; p = 0.014) for cervical stenosis when the height was greater than or equal to 20 mm, compared with the groups with or without stenosis.

This study was unable to prove the existence of a prognostic factor for cervical stenosis. However, it suggests that hemorrhagic complications of the immediate pre or postoperative period, which lead to the indication of suturing of the operative wound or its intense cauterization, might be consequences of cervical stenosis. We believe that one of the limitations of this study relates to the sample size and the small number of cases of stenosis, which may have led to beta error or type II error. In other words, it is possible that there are differences that were not demonstrated to be statistically significant at the 5% level. We hope that these results might lead to future studies with the objective of avoiding the occurrence of cervical stenosis following electrosurgical conization of the cervix.

## CONCLUSIONS

The crude incidence of cervical stenosis was 7.66%, and the incidence density was 3.3/1000. There was no prognostic feature associated with cervical stenosis.
